# Case Report: Anterior Spinal Cord Ischemia Following Embolization of Cerebellar Arteriovenous Malformation: An Illustrative Case and Review of Spinal Cord Vascular Anatomy

**DOI:** 10.3389/fneur.2021.725065

**Published:** 2021-09-07

**Authors:** Yasaman Moazeni, Donald R. Cantrell, Jeffrey R. Clark, Ramez N. Abdalla, Ayush Batra, Michael C. Hurley, Sameer A. Ansari, Eric J. Russell, Ali Shaibani

**Affiliations:** ^1^Department of Radiology, Northwestern University Feinberg School of Medicine, Chicago, IL, United States; ^2^Department of Neurology, Northwestern University Feinberg School of Medicine, Chicago, IL, United States; ^3^Department of Neurological Surgery, Northwestern University Feinberg School of Medicine, Chicago, IL, United States

**Keywords:** stroke, spinal cord ischemia, arteriovenous malformation, embolization, vascular diseases, spinal cord

## Abstract

Spinal cord ischemia (SCI) is a rare entity with high mortality and morbidity which can arise from causes such as atherosclerosis, aortic dissection or aneurysm, thromboembolic events or systemic hypotension, and is a potential complication of spinal surgery. Published literature contains very few reports of SCI as a complication of intracranial interventions, highlighting the uncommon nature of SCI in these circumstances. We report the occurrence of anterior SCI in a 69-year-old patient following successful embolization of a cerebellar arteriovenous malformation (AVM), marked by upper extremity weakness, lower extremity paraplegia, loss of bladder and bowel control, and hypercapnic respiratory failure requiring mechanical ventilation. Magnetic resonance imaging (MRI) demonstrated upper cervical diffusion restriction and T2/STIR hyperintensity. Unusually, SCI occurred in this case without intraprocedural catheter wedging or obvious flow limitation, prolonged procedure time, hypercoagulable state, or general hypotension. We review previous cases in the literature as well as spinal cord vascular anatomy, and discuss the possible etiologies of this complication. Spinal cord ischemia could be a very rare complication of neuroendovascular procedures even in the absence of warning signs and should be carefully evaluated in patients with suspected neurologic symptoms after such procedures.

## Introduction

Spinal cord ischemia (SCI), a rare entity with high mortality and morbidity, can be related to various etiologies including atherosclerosis, aortic dissection/aneurysm, cardiac emboli, and systemic hypotension. It mostly involves the anterior spinal cord (1–3). The incidence of SCI as a complication of intracranial interventions is very rare and has only been described in three case reports (3–5). Herein, we report the fourth known case of such complication following a neurointerventional procedure, an anterior SCI subsequent to embolization of a cerebellar arteriovenous malformation (AVM), and discuss the possible etiologies of this complication to highlight the importance of careful assessment of patients with suspected neurologic symptoms for SCI after neuroendovascular procedures.

## Case Description

The patient was a 69-year-old female with a past medical history of atrial fibrillation, hypertension, hyperlipidemia, and hypothyroidism. She denied any family history of neurovascular disease, AVM, or aneurysm. A right cerebellar hemispheric AVM was discovered incidentally during workup for dizziness. She had undergone neurologic work-up for intermittent vertigo 2 years before the admission, which in turn had resulted in the discovery, on brain MRI, of a right cerebellar hemispheric lesion, thought to represent a cavernous malformation (CM). At that time, she did not have any complaints of headache, motor or sensory disturbances, or loss of bladder or bowel control. Her neurologic evaluation was intact. Thus, she was being followed up by brain MRI and neurologic examination annually. The repeat MRI brain during her second year of follow up was interpreted as demonstrating an AVM rather than a CM, and she was scheduled for cerebral angiography to confirm the diagnosis.

**Figure 1:**
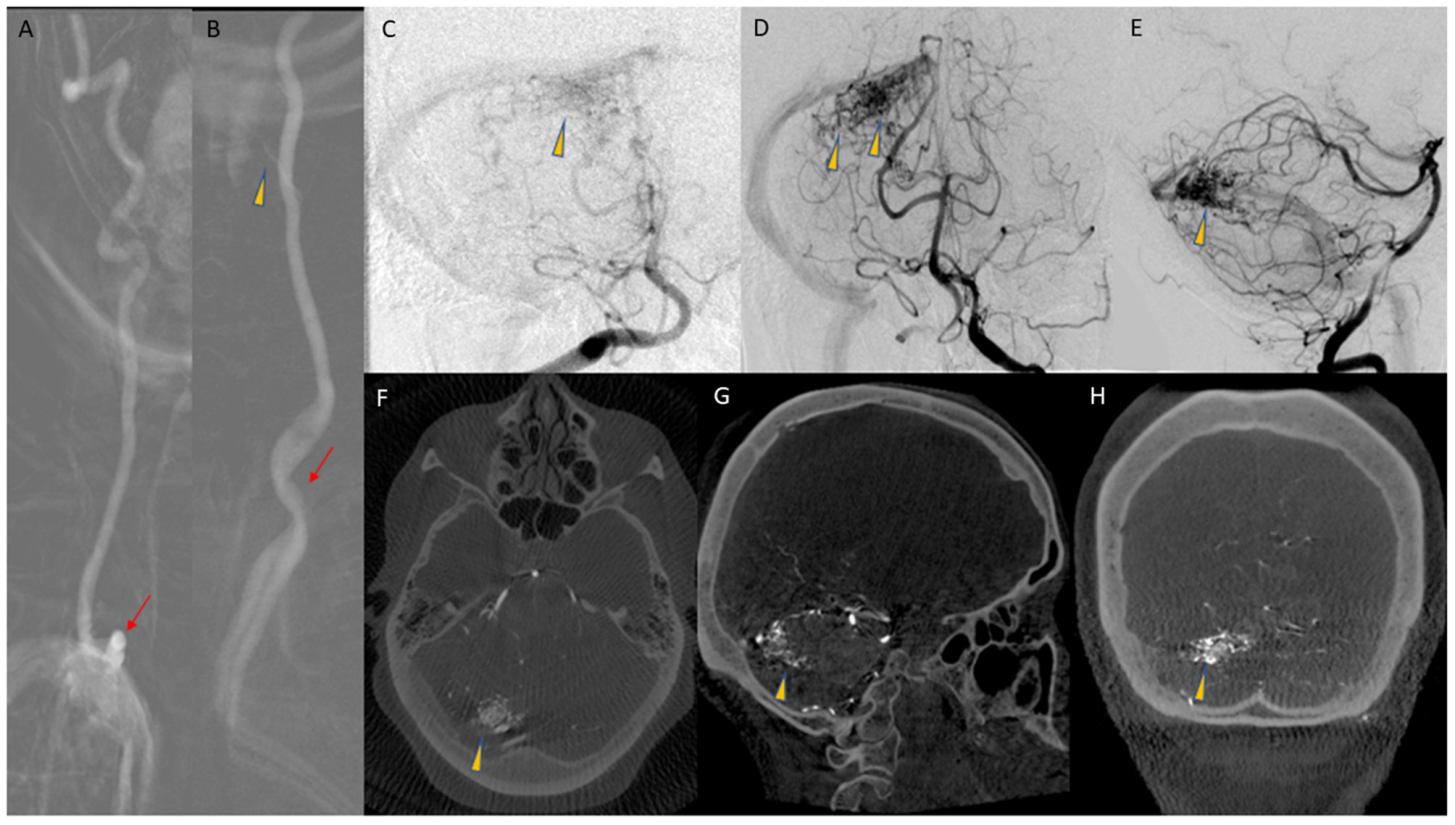
**(A)** AP roadmap image of co-dominant right VA, with tortuous origin (arrow) from the subclavian artery. **(B)** AP roadmap of left VA origin (arrow) and proximal cervical course of the artery. The artery of cervical enlargement is also seen (arrowhead). **(C)** AP angiogram from a right VA injection, with opacification of the AVM nidus (arrowhead), with competitive flow from the left VA. **(D,E)** AP and lateral arterial phase images from a left VA injection, demonstrating the AVM nidus (arrowheads) in the superior aspect of the right cerebellar hemisphere. **(F–H)** Reconstruction of 3D DSA data in axial **(F)**, Sagittal **(G)**, and coronal **(H)** planes, demonstrating the AVM nidus (arrowheads).

Angiography revealed a 2.5 cm pial AVM in the superomedial right cerebellar hemisphere, abutting the tentorium. The AVM was supplied primarily through branches of a hypertrophied right superior cerebellar artery (SCA), with lesser arterial supply provided by distal branches of the right posterior inferior cerebellar artery (PICA) (Figure 1). A single short superficial cerebellar vein drained the AVM into a right cerebellar tentorial vein, and then into the right transverse sinus. Two small nidal aneurysms were also identified, the largest of which measured 1.8 mm in maximal dimension. Magnetic Resonance T2 gradient echo imaging demonstrated small foci of susceptibility in the region of the AVM, presumably reflecting small areas of chronic hemosiderin deposition, but there was no evidence of recent hemorrhage. The vertebral arteries were relatively co-dominant.

After neurosurgical consultation, surgical resection of the AVM was recommended, and pre-surgical embolization was requested to minimize the risk of intraoperative bleeding.

Embolization was performed under general anesthesia. Blood pressure, heart rate, and oxygen saturation were monitored intra-procedurally. The mean arterial pressure (MAP) averaged 94.5 mmHg (range 76–117 mmHg) and no hypoxia or hypotension occurred throughout the procedure. Using ultrasound guidance, a five French shuttle guide sheath was placed into the right common femoral artery. Heparin was administered intravenously with intermittent bolus administration to maintain therapeutic anticoagulation. Activated Clotting Time was monitored, with an average value of 201 and a baseline of 135.

**Figure 2:**
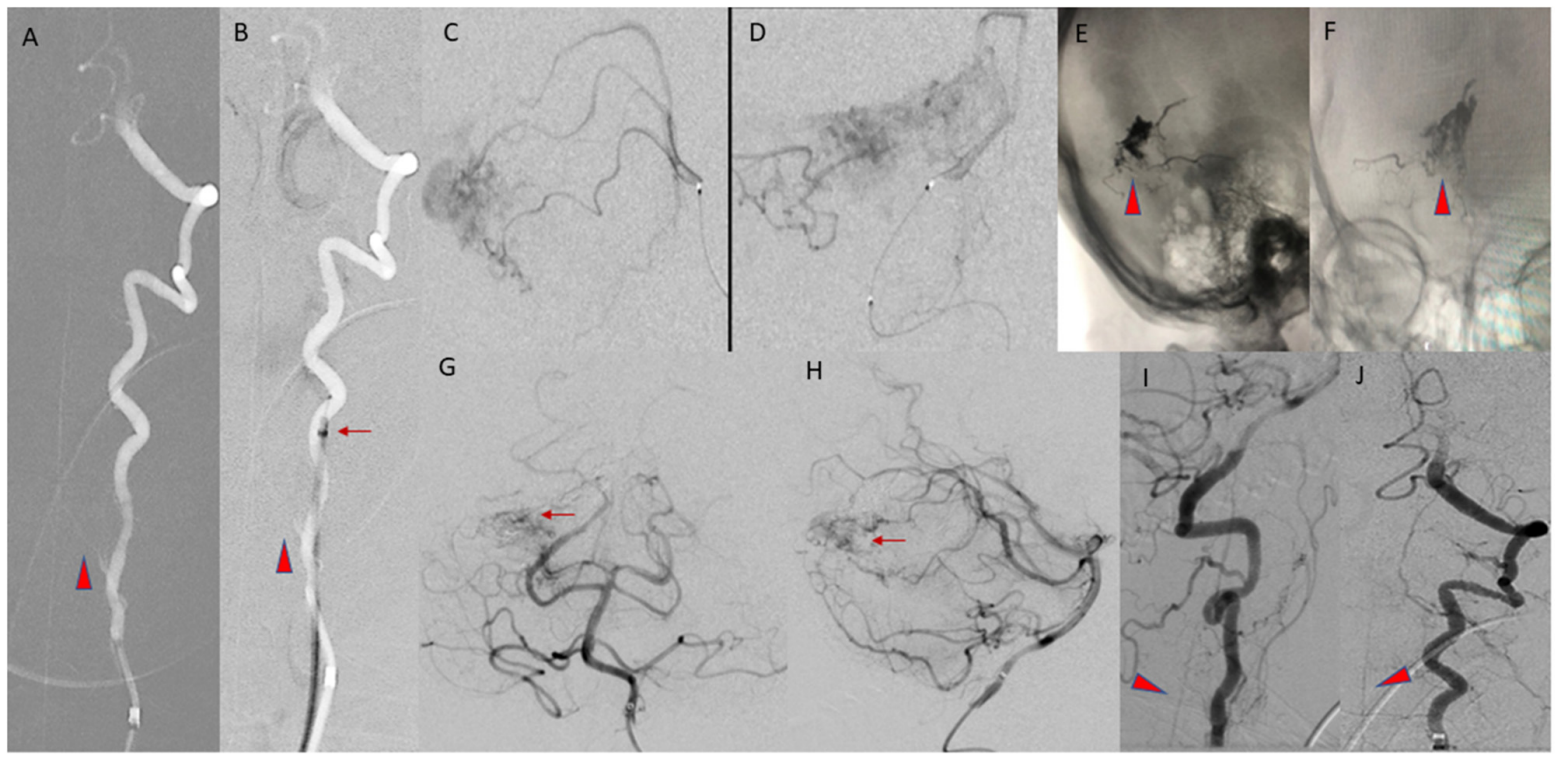
**(A,B)** AP Roadmap images of the left VA, before **(A)** and after **(B)** advancement of the five Fr guide sheath into the mid cervical left VA, distal to the origin of the artery of cervical enlargement (arrowheads). The position of the tip of the guide sheath is marked by the arrow. **(C,D)** Lateral and AP views of a right superior cerebellar artery microcatheter injection, during embolization of the AVM. **(E,F)** Final cast of the Onyx (arrowheads) within the AVM nidus. **(G,H)** Final AP and lateral arterial phase images of the posterior circulation, from a left VA injection, demonstrating the residual nidus (arrows). **(I,J)** Lateral and AP images from the final left VA injection prior to removal of the guide sheath, demonstrating patency of the ASA (arrowheads).

The sheath was advanced over a four French angled vertebral catheter into the proximal cervical left vertebral artery (VA). As noted on the diagnostic angiogram, the left VA had a variant origin, arising directly from the aortic arch, and was chosen for access due to marked tortuosity at the origin of the right VA. Five micrograms of intra-arterial verapamil was administered to prevent vasospasm, and the five French guide sheath was advanced to the mid-cervical left VA, beyond the origin of the artery of cervical enlargement, which was visualized on roadmap digital subtraction angiograms (DSA). The vertebral catheter was removed and DSA was performed through the sheath. There was good flow around the five French sheath without evidence of any contrast stagnation to suggest flow compromise or occlusion of the VA (Figure 2). The AVM was unchanged compared to the angiogram 2 months earlier.

Next, a Phenom Plus delivery catheter and Apollo microcatheter were advanced coaxially over a microwire through the five French sheath, with the Phenom Plus catheter terminating in the distal cervical left VA. An Apollo microcatheter was first taken distally into the dominant SCA branch supplying the AVM, and Onyx 34 was used to embolize a portion of the AVM nidus. The Apollo microcatheter was slowly removed, and the tip detached, remaining in the right SCA. Next, a new Apollo microcatheter was taken into a second SCA branch supplying the AVM, and additional Onyx was administered. This microcatheter was removed, without tip detachment. Finally, an SL-10 microcatheter was advanced into an inferior branch of the right SCA supplying the AVM, and a small amount of Onyx was administered before removing the microcatheter.

The remaining branches of the right SCA supplying the AVM were not easily catheterized. Embolization had resulted in an approximately 50% reduction in the size of the AVM nidus, so a decision was made to terminate the exam. Post-embolization intracranial and cervical angiography demonstrated patency of the arteries of the posterior circulation and the extracranial and intracranial segments of the left VA. The artery of cervical enlargement and the ASA remained patent at completion of the procedure. The sheath was removed, and hemostasis achieved. A Cone beam CT demonstrated expected post-procedural changes.

The patient was extubated and transferred to the intensive care unit. She was slow to awaken from general anesthesia, but was oriented, following commands, and moving all extremities antigravity immediately post-extubation.

On evaluation 3 h post-procedure, she was sleepy but easily aroused by voice, and could follow simple commands. Her neurologic examination at that time was intact. Additionally, she had bilateral upper extremity strength of at least 4/5 and intact light touch sensation throughout. Over the next 2 h, her condition mostly remained unchanged, with mild weakness of bilateral upper extremities on examination that was suspected to be limited by the element of effort and of position, given otherwise normal findings. She was reported to have general weakness at approximately 8 h after the procedure. Her situation did not improve after 2 h and required a repeat of examination and further evaluation.

**Figure 3:**
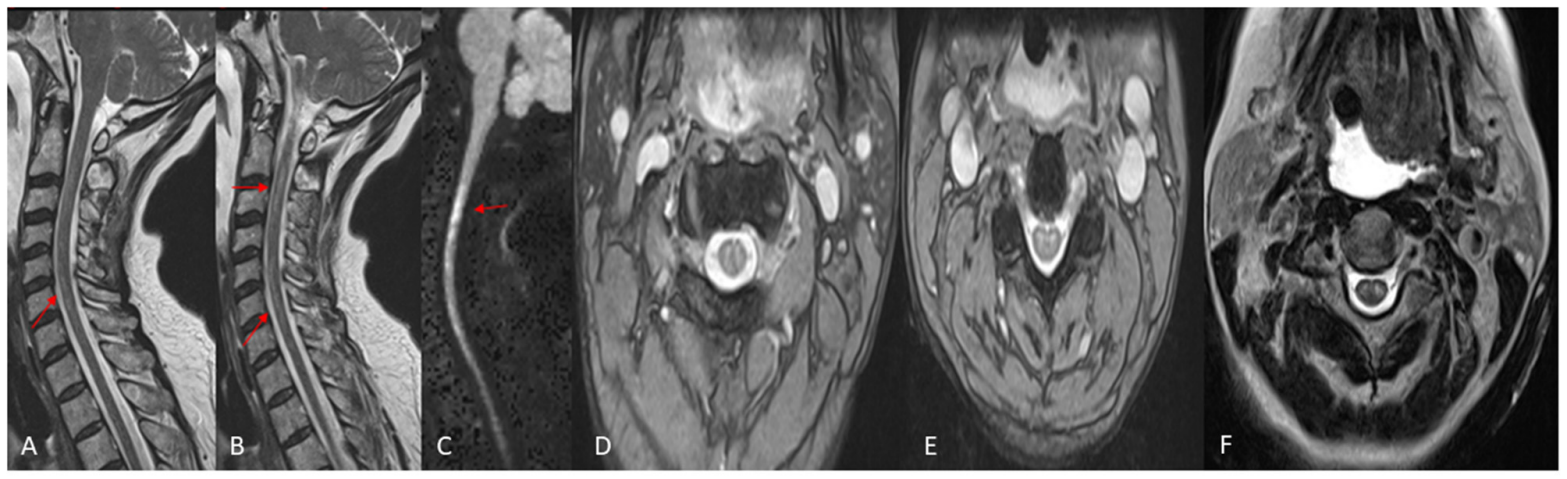
MRI Cervical Spine, performed the day following the embolization. **(A,B)** Sagittal T2W images demonstrating abnormal increased T2 signal in the spinal cord (arrows). **(C)** Sagittal Diffusion Weighted image demonstrating abnormal diffusion restriction in the Cervical Spinal cord (arrows). **(D,E)** Axial GRE T2W images demonstrating abnormal increased signal in the central Gray matter, or “owls eye” sign. **(F)** Axial TSE T2W image demonstrating the “owls eye” sign.

**Figure 4:**
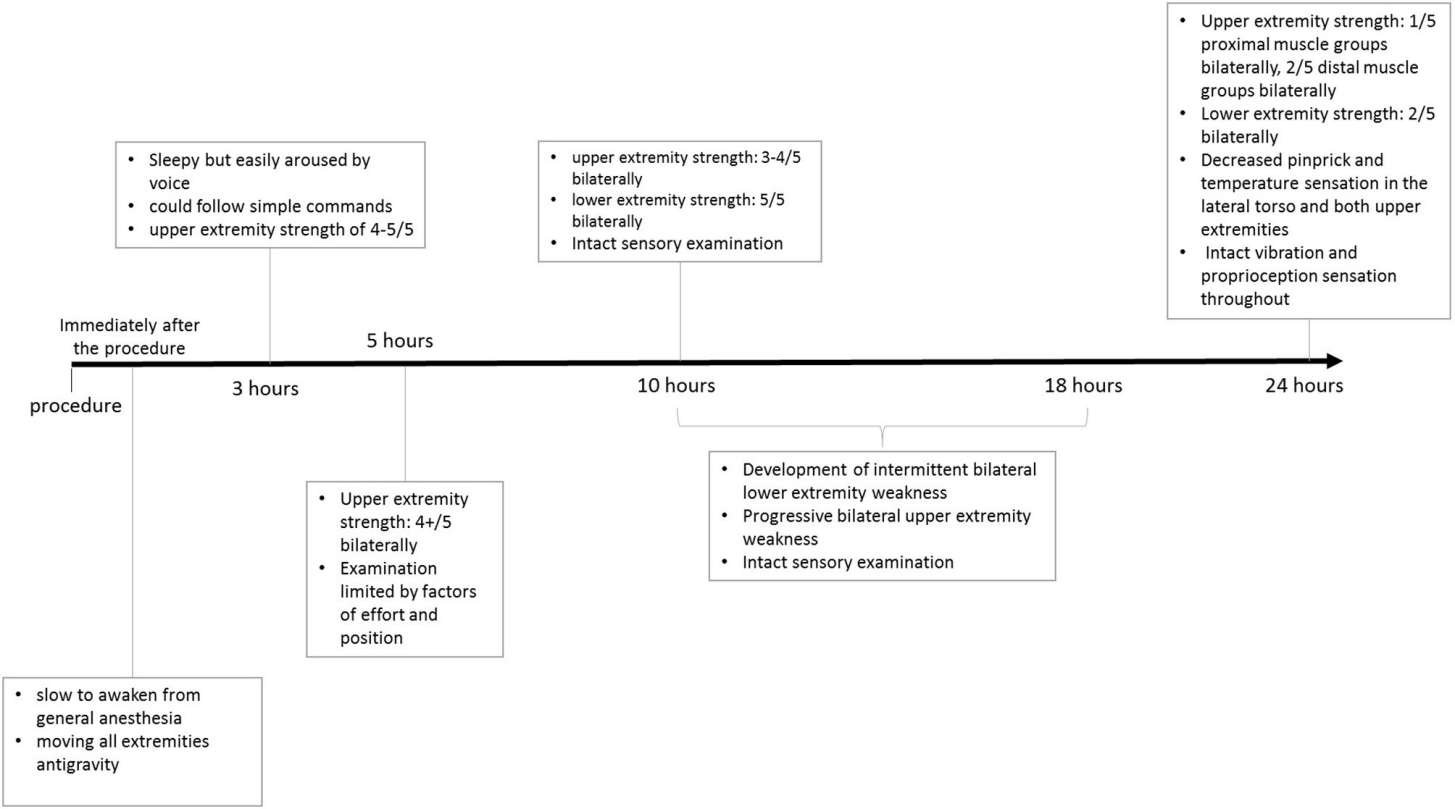
Timeline of events post-procedure.

Her examination, approximately 10 h post-embolization, revealed strength of 3–4/5 in both upper extremities. Her lower extremities strength remained 5/5, with normal pinprick and vibration sensory exam throughout. Her vital signs were within normal limits and stable, and her examination was otherwise unremarkable. However, concern for ischemia prompted a Magnetic Resonance Imaging (MRI) brain, which suggested diffusion restriction in the upper cervical spinal cord, although this was sub-optimally evaluated due to motion artifact. A cervical spine MRI was obtained next and demonstrated T2/STIR hyperintense signal at the C1 level, corresponding to the region of restricted diffusion reported on MRI of the brain, raising suspicion for possible cord infarction. The patient's upper extremity weakness continued to progress over the subsequent 8 h despite maintaining MAP >75 mmHg, and stable vital signs, and she developed new intermittent lower extremity weakness, but light touch sensation remained intact in all extremities. Clinical examination at this point was consistent with acute SCI. A second cervical MRI revealed more extensive abnormal signal centered at the C2 and C3 levels with associated cord expansion. Additionally, new signal abnormality had developed at the C5–C6 level, without associated cord expansion (Figure 3). Her limb weakness continued to progress. On evaluation 1 day post-procedure, she had a strength of 1/5 and 2/5 in proximal and distal muscle groups of both upper extremities, respectively, with a strength of 2/5 in both lower extremities. Pinprick and temperature sensation were decreased at the approximate level of C2–C4 throughout the lateral torso and both upper extremities, while vibration and proprioception remained intact in all extremities. These findings reinforced the diagnosis of acute SCI. Figure 4 demonstrates the timeline of the events within 24 h after the procedure. A second brain MRIat this time showed evidence of T2/FLAIR hyperintense signal and increased diffusion restriction in the right cerebellar hemisphere at the site of Onyx embolization. As a result of the SCI, she developed hypercapnic respiratory failure requiring urgent endotracheal intubation and mechanical ventilation. She eventually required tracheostomy placement for anticipated prolonged ventilator weaning along with percutaneous gastrostomy placement. She was successfully weaned off the ventilator and resumed oral intake by 3 weeks, and underwent acute inpatient rehabilitation for a total 4 months, and is now residing at home with significant assistance.

## Discussion

Arterial supply to the spinal cord can be divided into (1) the intrinsic arteries, located within the cord's substance, (2) the extrinsic arteries of the spinal canal, which travel longitudinally and circumferentially along the cord's surface, and (3) the radiculomedullary and radiculopial arteries, which enter the spinal canal from the extraspinal vasculature at variable segmental levels.

The intrinsic arteries of the spinal cord can be anatomically separated into central and peripheral systems. The central system is supplied by the sulco-commissural arteries, which arise from the anterior spinal artery (ASA) and supply the majority of the central gray matter. In this system, blood flows from the center of the spinal cord to the periphery. The peripheral intrinsic system is supplied by radial perforating arteries that originate from the pial network, a highly variable network of arteries that branch from both the ASA and posterior spinal artery (PSA) to encircle the spinal cord. The peripheral system primarily perfuses the white matter of the spinal cord, and through the PSAs supplies the dorsal columns of the gray matter.

The ASA, paired PSAs, and the circumferential pial network comprise the extrinsic arterial system of the spinal canal.

**Figure 5:**
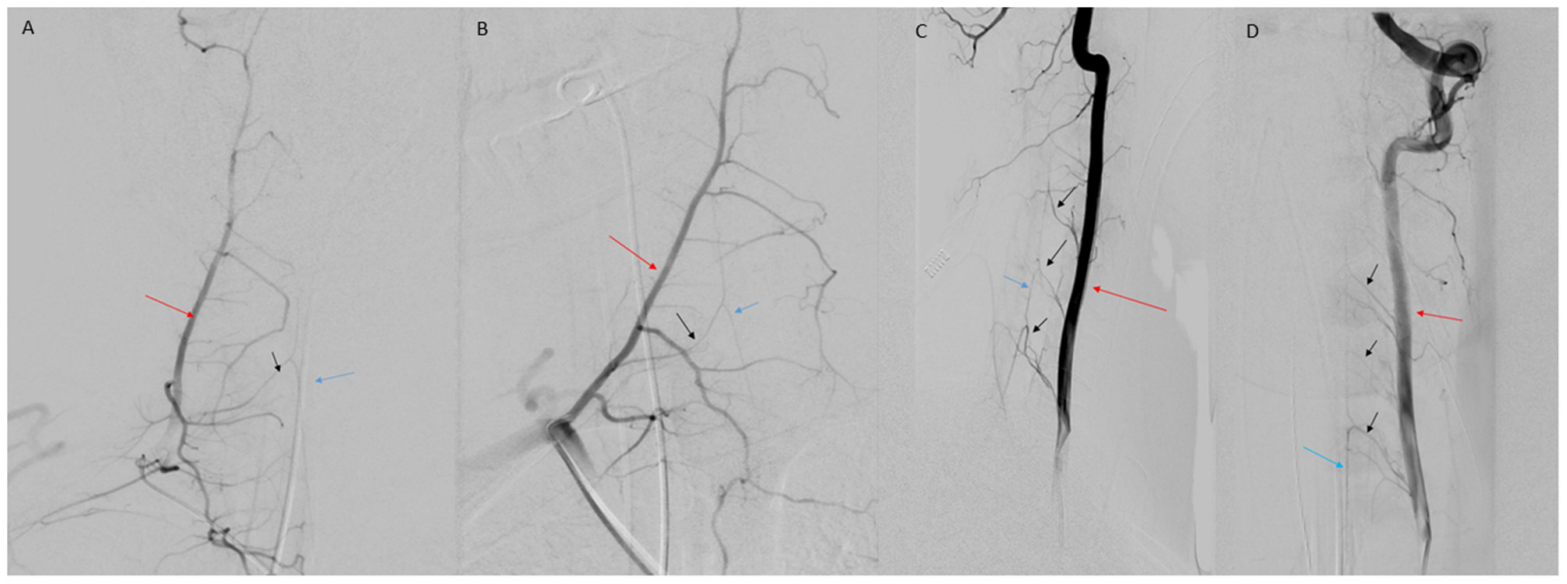
**(A)** AP view of right deep cervical artery (red arrow) injection demonstrating radiculomedullary feeder (black arrow) to the ASA (blue arrow). **(B)** Oblique view of deep cervical artery (red arrow) injection demonstrating radiculomedullary artery (black arrow) supply to the ASA (blue arrow). **(C)** Lateral view of VA (red arrow) injection demonstrating multiple cervical radiculomedullary feeders (black arrows) to the ASA (blue arrow). **(D)** AP view of left VA (red arrow) injection demonstrating multiple cervical radiculomedullary feeders (black arrows) to the ASA (blue arrow).

The ASA, the dominant artery of the spinal vasculature that supplies the anterior two-third**s** of the cord, is derived from branches arising from the intracranial segments of both VAs, and travels inferiorly along the ventral surface of the spinal cord, although in some cases, the ASA can arise from the intracranial segment of a single VA. The ASA is further supplied by anastomoses to a variable number of radiculomedullary arteries (Figure 5) throughout the cervical, thoracic, and lumbar spine, which range from 6 to 10 in number (6). The paired PSAs originate either from the intracranial VAs proximal to the PICAs or from the proximal PICAs. These arteries travel longitudinally and discontinuously along the dorsolateral surface of the cord, with variable contributions from radiculopial segmental branches.

Radiculomedullary and radiculopial arteries supply the ASA and the PSAs, respectively (1, 7, 8). The largest cervical radiculomedullary artery is often called the artery of cervical enlargement (7, 9). The number of sizable radiculomedullary and radiculopial arteries supplying the cervical cord varies. Haller reported as many as 11 radiculomedullary arteries and one radiculopial artery in the cervical spine (10), while Adamkiewicz observed three sizable radiculomedullary branches and one or two radiculopial arteries (11). Subsequent cadaveric studies reported a variable range of radiculomedullary arteries between 1 and 6 (10). Chakravorty found that most commonly, two or three radiculomedullary arteries >250 μm in size contribute to the ASA in the cervical spine, typically arising from C4, C5, or C6 radicular arteries. He also reported that the number of major radiculomedullary vessels in the cervical spine measuring >500 μm was never more than two, and only a single major radiculomedullary branch of this size was present in 12 of 31 spinal cords studied (10). The pial network, ASA, and PSAs create a rich vascular supply to the cervical spine, decreasing the likelihood of ischemia in most patients. However, due to the high degree of anatomic variability, in some individuals a paucity of radiculomedullary arteries can increase the risk of anterior SCI when provoked by local or systemic hemodynamic insufficiency (4).

Spinal cord ischemia has various manifestations including paraplegia, sensory deficits and urinary incontinence (12). Anterior SCI can present with bilateral motor dysfunction and pain and temperature sensation disturbance below the level of infarction with preserved posterior column pathways, supporting vibration and proprioception (13). Spinal cord ischemia has been reported following numerous surgeries including spinal surgeries as well as aortic and cardiac interventions (2, 4, 14, 15), but onset after an intracranial intervention is very rare, reported only by a few publications (3–5). In all prior reports, including our own, infarction occurred in the cervical region.

A case of unilateral posterior cervical SCI following vertebral angioplasty was attributed to flow reversal in the VA due to a persistent stenosis in the VA, resulting in hypoperfusion or thromboembolism in the ipsilateral PSA (3).

In three cases of cervical SCI following embolization of basilar tip aneurysms involving the use of six French or seven French guide catheters, the authors posited that wedging of the catheter in the VA may have resulted in thromboembolic occlusion or flow restriction of radiculomedullary branches of the VA supplying the cervical spinal cord. They proposed downsizing the guide catheter to five French as a possible solution. We utilized a five French guide sheath (similar outer diameter to a seven French guide catheter), without angiographic evidence of “wedging” or flow arrest during the procedure.

Hypotension resulting in infarction at watershed areas of the cervical spinal cord located in between radiculomedullary arterial anastomoses is unlikely in this case, as the MAP was stable throughout the embolization with no evidence of hypotension. Thromboembolic events in the VA, ASA, or the artery of cervical enlargement are unlikely as these arteries were patent in the post-embolization left VA angiogram, and additionally, the tip of the five Fr sheath was beyond the origin of the artery of cervical enlargement.

This is an unusual case of successful embolization of a cerebellar AVM through the posterior circulation complicated by cervical SCI, without catheter wedging/flow arrest, prolonged procedure time, hypercoagulable state, or general hypotension. It is likely that with the use of a five French sheath in the left VA, which is comparable in diameter to a six or seven French guide catheter, and even in the absence of catheter wedging and flow limitation, there was enough reduction in perfusion pressure to the ASA through the artery of cervical enlargement to cause SCI. We speculate that this patient and the other reported similar cases may have occurred in patients who happened to have a reduced number of radiculomedullary suppliers to the ASA. Spinal cord ischemia can be a very rare complication of neuroendovascular procedures even in the absence of warning signs and should be carefully evaluated in patients with suspected neurologic symptoms after such procedures.

Given the rarity of SCI relative to the large numbers of endovascular procedures where the VA is accessed with guide sheaths and catheters, it remains unclear whether one can recommend a thorough assessment of spinal cord arterial supply prior to every endovascular intervention in the posterior circulation. Most anatomic series and textbooks indicate an average of 2–3 radiculomedullary arteries supplying the ASA at the level of cervical spinal cord. However, the high degree of variation in the number of radicullomedullary arteries, puts patients with paucity of radicullomedullary arteries at a greater risk for developing spinal ischemic events following intracranial endovascular interventions in the posterior circulation. Although rare, given the high morbidity and mortality of such complication, it seems reasonable to recommend a thorough assessment of spinal cord arterial supply before endovascular interventions, for patients in whom the artery of cervical enlargement originates from the VA that is planned to be accessed via a guide sheath or catheter. Also, consistent with prior reports we suggest downsizing from a 5 F guide sheath might be a solution to reduce the risk of this rare and devastating complication in such patients.

## Data Availability Statement

The original contributions presented in the study are included in the article/supplementary material, further inquiries can be directed to the corresponding author/s.

## Ethics Statement

This study was granted a waiver of consent by the Northwestern University Institutional Review Board (STU00213955).

## Author Contributions

YM, DRC, and RNA collected the patient's material. RNA, AB, and AS were responsible for clinical examination of the patient. RNA, MCH, SAA, EJR, and AS reviewed patient's imaging. YM, DRC, JRC, and AS wrote the manuscript. DRC, JRC, AB, MCH, SAA, and EJR edited the manuscript. All authors contributed to the article and approved the submitted version.

## Funding

Northwestern Open Access Fund provided by Northwestern University Libraries to cover open access publication fees.

## Conflict of Interest

The authors declare that the research was conducted in the absence of any commercial or financial relationships that could be construed as a potential conflict of interest.

## Publisher's Note

All claims expressed in this article are solely those of the authors and do not necessarily represent those of their affiliated organizations, or those of the publisher, the editors and the reviewers. Any product that may be evaluated in this article, or claim that may be made by its manufacturer, is not guaranteed or endorsed by the publisher.

## Abbreviations

AP: anteroposterior
ASA: anterior spinal artery
AVM: arteriovenous malformation
DSA: digital subtraction angiograms
MAP: mean arterial pressure
MRI: magnetic resonance imaging
PICA: posterior inferior cerebellar artery
SCI: spinal cord ischemia
SCA: superior cerebellar artery
VA: vertebral artery.

## References

[1] Cheshire WP Santos CC Massey EW Howard JF Jr. Spinal cord infarction: etiology and outcome. Neurology. (1996) 47:321–30. 10.1212/wnl.47.2.321 8757000

[2] Kaya A Yildiz Z Nurkalem Z. Spinal cord infarction as a complication of percutaneous coronary intervention. Spinal Cord. (2014) 52(Suppl 2):S5–7. 10.1038/sc.2014.80 25082382

[3] Elzamly K Nobleza C Parker E Sugg R. Unilateral upper cervical posterior spinal cord infarction after a neuroendovascular intervention: a case report. Case Rep Neurol Med. (2018) 2018:5070712. 10.1155/2018/5070712 30073102 PMC6057308

[4] Matsubara N Miyachi S Okamaoto T Izumi T Asai T Yamanouchi T. Spinal cord infarction is an unusual complication of intracranial neuroendovascular intervention. Interv Neuroradiol. (2013) 19:500–5. 10.1177/159101991301900416 24355157 PMC3902752

[5] Iwahashi H Fujita A Tanaka H Ikeda M Morikawa M Kohmura E. Spinal cord infarction after successful coil embolization of recurrent basilar bifurcation aneurysm: a case report. J Neuroendovasc Ther. (2018) 12:398–403. 10.5797/jnet.cr.2017-0117

[6] Bosmia AN Hogan E Loukas M Tubbs RS Cohen-Gadol AA. Blood supply to the human spinal cord: part I. Anatomy and hemodynamics. Clin Anat. (2015) 28:52–64. 10.1002/ca.22281 23813725

[7] Martirosyan NL Feuerstein JS Theodore N Cavalcanti DD Spetzler RF Preul MC. Blood supply and vascular reactivity of the spinal cord under normal and pathological conditions. J Neurosurg Spine. (2011) 15:238–51. 10.3171/2011.4.Spine10543 21663407

[8] Richard S Abdallah C Chanson A Foscolo S Baillot PA Ducrocq X. Unilateral posterior cervical spinal cord infarction due to spontaneous vertebral artery dissection. J Spinal Cord Med. (2014) 37:233–6. 10.1179/2045772313y.0000000125 24090478 PMC4066433

[9] Turnbull IM. Blood supply of the spinal cord: normal and pathological considerations. Neurosurgery. (1973) 20:56–84. 10.1093/neurosurgery/20.cn_suppl_1.56 4762823

[10] Chakravorty BG. Arterial supply of the cervical spinal cord (with special reference to the radicular arteries). Anat Rec. (1971) 170:311–29. 10.1002/ar.1091700308 5088404

[11] Adamkiewicz A. Die blutgefasse des menschlichen ruckenmarkes oberfiache. Sitz d Wiss in Wien Math Natur Klass. (1882) 85:101–35.

[12] Alexander J Yohannan T Abutineh I Agrawal V Lloyd H Zurakowski D. Ultrasound-guided femoral arterial access in pediatric cardiac catheterizations: a prospective evaluation of the prevalence, risk factors, and mechanism for acute loss of arterial pulse. Catheter Cardiovasc Interv. (2016) 88:1098–107. 10.1002/ccd.26702 27535615

[13] Weidauer S Nichtweiss M Lanfermann H Zanella FE. Spinal cord infarction: MR imaging and clinical features in 16 cases. Neuroradiology. (2002) 44:851–7. 10.1007/s00234-002-0828-5 12389137

[14] Blankenship JC Mickel S. Spinal cord infarction resulting from cardiac catheterization. Am J Med. (1989) 87:239–40. 10.1016/s0002-9343(89)80710-7 2757065

[15] Shlobin NA Raz E Shapiro M Clark JR Hoffman SC Shaibani A. Spinal neurovascular complications with anterior thoracolumbar spine surgery: a systematic review and review of thoracolumbar vascular anatomy. Neurosurg Focus. (2020) 49:E9. 10.3171/2020.6.focus20373 32871559

